# Directly Observed Treatment Short-course (DOTS) for tuberculosis control program in Gambella Regional State, Ethiopia: ten years experience

**DOI:** 10.1186/1756-0500-7-44

**Published:** 2014-01-20

**Authors:** Solomon Sisay, Belete Mengistu, Woldaregay Erku, Desalegn Woldeyohannes

**Affiliations:** 1 Department of Clinical, John Hopkins University-TSEHAI Project, P.O. Box 5606, Addis Ababa, Ethiopia; 2 Directorate of Pastoralist Health Promotion and Disease Prevention, Federal Ministry of Health, P.O. Box 1234, Addis Ababa, Ethiopia; 3 Department of Tropical and Infectious Diseases, Aklilu Lemma Institute of Pathobiology Addis Ababa University, P.O. Box 1176, Addis Ababa, Ethiopia; 4 Department of Public Health, School of Medicine and Health Sciences, Addis Ababa Science and Technology University, P.O. Box 16417, Addis Ababa, Ethiopia

**Keywords:** Gambella Regional State, Case detection rate, Directly observed treatment short-course, Treatment success rate, Tuberculosis

## Abstract

**Background:**

Tuberculosis is still the leading cause of illness in the world which accounted for 2.5% of the global burden of disease, and 25% of all avoidable deaths in developing countries. The aim of study was to assess impact of DOTS strategy on tuberculosis case finding and treatment outcome in Gambella Regional State, Ethiopia from 2003 up to 2012 and from 2002 up to 2011, respectively.

**Methods:**

Health facility-based retrospective study was conducted. Data were collected and reported in quarterly basis using WHO reporting format for TB case finding and treatment outcome from all DOTS implementing health facilities in all zones of the region to Federal Ministry of Health.

**Results:**

A total of 10024 all form of TB cases had been registered between the periods from 2003 up to 2012. Of them, 4100 (40.9%) were smear-positive pulmonary TB, 3164 (31.6%) were smear-negative pulmonary TB and 2760(27.5%) had extra-pulmonary TB. Case detection rate of smear-positive pulmonary TB had increased from 31.7% to 46.5% from the total TB cases and treatment success rate increased from 13% to 92% with average mean value of being 40.9% (SD = 0.1) and 55.7% (SD = 0.28), respectively for the specified year periods. Moreover, the average values of treatment defaulter and treatment failure rates were 4.2% and 0.3%, respectively.

**Conclusion:**

It is possible to achieve the recommended WHO target which is 70% of CDR for smear-positive pulmonary TB, and 85% of TSR as it was already been fulfilled the targets for treatments more than 85% from 2009 up to 2011 in the region. However, it requires strong efforts to enhance case detection rate of 40.9% for smear-positive pulmonary TB through implementing alternative case finding strategies.

## Background

Tuberculosis (TB) is a bacterial disease caused mainly by mycobacterium tuberculosis, which is an acid fast and rod shaped bacillus [1]. TB is still the leading cause of illness worldwide which accounted for 2.5% of global burden of disease. About a third of the world’s population is estimated to be infected with tubercle bacilli, and hence at risk of developing active disease [2]. According to world health organization (WHO) global TB report in 2011, there were globally an estimated 9 million incident cases of TB. Of which, 1.2 million were among people living with HIV. Annual number of deaths due to TB was 1.6 million, including 195000 patients infected with HIV [3].

In developing countries, TB comprised 25% of all avoidable adult deaths. It is estimated that nearly one million (11%) of the total TB cases were children whose age less than 15 years. About 26% of TB incident cases occurred in Africa in 2010 [2]. Moreover, 22 high burden countries (HBCs) accounted for approximately 80% of the estimated number of new all form of TB cases which rising worldwide on the same year. In Africa, those countries with high rates of TB/HIV co-infection are the main focus of intensified efforts in directly observed treatment short-course (DOTS) expansion. Ethiopia was ranked seventh among the world 22 high burden countries (HBCs) [3].

The first national population based TB prevalence survey was conducted by Ethiopian Health Nutrition and Research Institution (EHNRI) from 2010 up to 2011 which revealed that the prevalence of smear positive TB among adults and all age group were found to be 108 cases and 63 cases per 100000 populations, respectively. The prevalence of bacteriologically confirmed TB was found to be 156 cases per 100000 populations, and the prevalence of all forms of TB in Ethiopia was estimated to be 240 cases per 100000 populations [4].

According to WHO global TB report in 2012, which considered the findings from the national TB prevalence survey, there were an estimated 220000 (258 cases per 100000 populations) incident cases of TB in Ethiopia in 2011. The same report mentioned that the prevalence of TB was estimated to be 200000 (237 cases per 100000 populations). Additionally, there were an estimated 15000 deaths (18 deaths per 100000 populations) due to TB, excluding HIV related deaths in the country [5]. Hospital statistics data of federal ministry of health (FMoH) revealed that tuberculosis is the leading cause of morbidity, the third cause of hospital admission (after deliveries and malaria), and the second cause of death in Ethiopia after malaria [2].

Hence, FMoH adopted DOTS strategy for TB control program in 2000 which was piloted before in a few areas of the country [6]. Then, a number of similar researches were conducted in some parts of the country for assessing experience of DOTS program for TB control in which the findings revealed that there were more gaps in organizational issues such as misuse and under use of TB registration book, challenges in follow-up of TB patients, low case detection of TB and increased rate of treatment defaulter [7].

Even though it is critical to evaluate the impact of DOTS program, more studies still remained to be carried out at different level of programmatic management including health facilities which provide direct treatment service to the community. Therefore, the study aimed at evaluating impact of DOTS program in Gambella Regional State on TB case finding and treatment outcome from 2003 up to 2012 and from 2002 up to 2011, respectively.

## Methods

### Study design

Health facility-based retrospective data was collected for TB cases who registered during the study period from 2003 up to 2012 and for their treatment outcome in Gambella Regional State from 2002 up to 2011.

### Study area

Gambella Regional State consisted of 4 administrative zones, 8 districts and 7 towns which was far 777 km from west of capital city, Addis Ababa. Rural parts of the region had 174 kebeles, while urban parts had 8 kebeles. Size of population in the region, for the year 2007 national census, was about 307096. Out of which, 159787 (52%) were males and 147309 (48%) females [8]. DOTS program was initiated in the region in 1993, and in 2011, DOTS coverage among public facilities like hospitals and health centers reached 100% and 75%, respectively while, it was only 11% among health posts. Currently, the program is being implemented in 1 government hospital, 21 health centers (17 governments and 4 NGOs), 7 health posts, 9 lower clinics (8 governments and 1 NGO) [9].

### Inclusion and exclusion criteria

All forms of TB cases who registered during the study period were included in the study. Treatment outcome of extra-pulmonary tuberculosis and smear-negative pulmonary tuberculosis cases were excluded as treatments outcome mainly focuses on smear-positive pulmonary tuberculosis cases due to their infectiousness than other forms of TB.

### Data collection procedures

Data were collected by world health organization (WHO) standardized reporting formats for case detection and treatment outcome. Reports from all zones in the region were collected by trained data collectors and investigators. Data were first collected from health facilities (HF) where TB focal persons compiled the data and reported it on quarterly basis about all TB patients entered into TB clinic thereby assigning a unique TB registration number for each TB patients and submitted the report to zonal TB focal persons who were responsible for compiling zonal summary, and in turn, the zonal TB focal persons submitted report to regional TB program officer. Regional TB program officer checked completeness, quality and accuracy of reports. Then, data were analyzed and interpreted, and sent as compiled report to the office of national tuberculosis and leprosy control program (NTLCP) office, federal ministry of health (FMoH).

### Data analysis

Data which were collected and reported from standardized WHO formats were analyzed and interpreted using SPSS version 17.0 packages. Data were summarized using frequencies, percentages and standard deviations including for mean values of variables like case detection rate, treatment success rate, death rate and defaulter rate.

### Data validation

Federal Ministry of Health (FMoH) used health management information system (HMIS) for all health program recording and reporting, which consisted of its own unique data collection book and reporting format. Data obtained by WHO reporting formats were crosschecked for the data obtained in HMIS for maintaining consistency of information within the study period.

### Operational definitions

### Case definitions

#### Case detection rate

Percentage of smear-positive TB cases detected among the total number of TB cases estimated to occur.

#### New case

A patient who has never had treatment for TB or has been on anti-TB treatment for less than four weeks.

#### Relapse

A patient who has been declared cured or treatment completed from any form of TB in the past but found to be smear-positive or culture positive.

#### Return after default

A patient who had previously been recorded as defaulted from treatment and returns to the health service with smear-positive sputum.

### Treatment out come

#### Defaulter

A patient who has been on treatment for at least 4 weeks and whose treatment was interrupted for 8 or more consecutive weeks.

#### Transfer Out (TO)

A patient who has started treatment and has been transferred to another health facility and for whom treatment outcome is not known at the time of evaluation.

#### Cured

A patient who is sputum smear-negative one month prior to the completion of treatment and on at least one previous occasion (usually at the end of the 2nd or 5th month).

#### Treatment completed

A patient who has completed treatment but in whom smear result are not available at or one month prior to the completion of treatment.

#### Treatment failure

A patient who remained smear positive or became again smear positive at the end of five months or later after commencing treatment.

#### Died

A patient who dies for any reason during the course of treatment.

#### Treatment success rate

A sum of TB cases who completed treatment and who declared cured.

### Ethical considerations

Ethical clearance was obtained from Institutional Review Board of School of Medicine and Health Science, AASTU and Ethical Clearance Committee of Gambella Regional Health Bureau.

## Result

**Figure 1:**
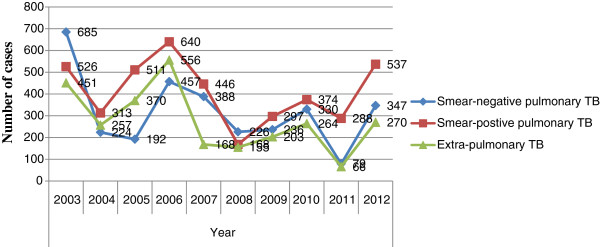
Trend of case finding for all forms of TB cases in Gambella Regional State from 2003 up to 2012.

A total of 10024 cases were registered with all forms of TB for the past 10 years (Table 1). Out of which, 4100(40.9%) were smear-positive pulmonary TB, 3164 (31.6%) smear-negative pulmonary TB, and 2760 (27.5%) extra-pulmonary TB. Among all forms of TB, 5615 (56%) were male patients and 4409 (44%) females. Trends of case finding for all forms of TB cases were shown for the decade (Figure 1).

**Table 1:** Case notifications of all forms of TB by sex in Gambella Regional State from 2003 up to 2012

	**Smear-positive PTB**				**Smear-negative PTB**			**Extra-pulmonary PTB**			**All forms of TB**		
**Year**	**M (%)**	**F (%)**	**Total (%)**	**CDR (%)**	**M (%)**	**F (%)**	**Total (%)**	**M (%)**	**F (%)**	**Total (%)**	**M**	**F**	**Grand total**
2003	295 (17.7)	231 (13.9)	526 (31.7)	31.7	385 (23.1)	300 (18.1)	685 (41.2)	253 (15.2)	198 (12)	451 (27.1)	930	732	1662
2004	175 (22)	138 (17.3)	313 (39.4)	39.4	126 (15.9)	98 (12.5)	224 (28.2)	44 (18.1)	113 (14.2)	257 (32.4)	445	349	794
2005	286 (26.7)	225 (21)	511 (47.6)	47.6	108 (10.1)	84 (7.8)	192 (17.9)	207 (19.3)	163 (15.2)	370 (34.5)	601	472	1073
2006	338 (20.5)	302 (18.3)	640 (38.7)	38.7	256 (15.5)	201 (12.2)	457 (27.6)	312 (18.9)	244 (14.8)	556 (33.6)	926	727	1653
2007	250 (25)	196 (19.6)	446 (44.5)	44.5	217 (21.7)	170 (17.1)	388 (38.7)	94 (9.4)	74 (7.4)	1689 (16.8)	561	441	1002
2008	91 (16.6)	77 (14)	168 (30.6)	30.6	127 (23.1)	99 (18)	226 (41.2)	87 (15.9)	68 (12.4)	155 (28.2)	308	241	549
2009	194 (26.4)	103 (14)	297 (40.4)	40.4	132 (18)	104 (14.1)	236 (32.1)	114 (15.5)	89 (12.1)	203 (27.6)	412	324	736
2010	189 (19.5)	185 (19.1)	374 (38.6)	38.6	185 (19.1)	145 (15)	330 (34.1)	148 (15.3)	116 (12)	264 (27.3)	542	426	968
2011	178 (41.1)	110 (25.4)	288 (66.5)	66.5	44 (10.2)	35 (8.1)	79 (18.2)	37 (8.5)	29 (6.7)	66 (15.2)	243	190	433
2012	301 (26.1)	236 (20.5)	537 (46.5)	46.5	194 (16.8)	153 (13.3)	347 (30.1)	151 (13.1)	119 (10.3)	270 (23.3)	645	509	1154
Total	2297 (22.9)	1803 (18)	4100 (40.9)	40.9	1773 (17.7)	1391 (13.9)	3164 (31.6)	1547 (15.4)	1213 (12.1)	2760 (27.5)	5615 (56)	4409 (44)	10024

**Figure 2:**
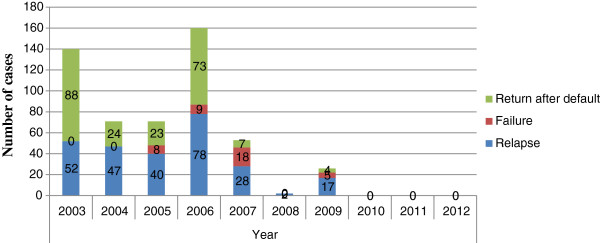
Number of smear-positive re-treatment case finding of Gambella Regional State from 2003 up to 2012.

**Figure 3:**
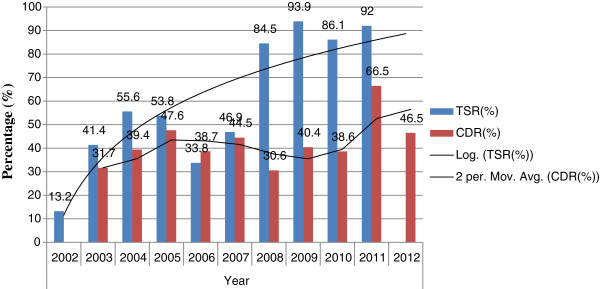
Trend of case detection rate and treatment success rate of smear-positive pulmonary TB in Gambella Regional State from 2002 up to 2012.

Among the total smear-positive pulmonary TB cases (4100), more new smear-positive pulmonary TB cases were reported each year than re-treatment smear-positive pulmonary TB cases (defaulter, failure and relapse) in the region. There were 1433 (35%) new smear-positive patients while 264 (6.4%) were relapse, 40 (0.9%) were failure and 219 (5.3%) were returned after default cases in the study period (Figure 2). Moreover, there were an increased number of males 123 (3%) who returned after default as compared females 96 (2.3%). Case detection rate of smear-positive pulmonary TB had increased from 31.7% to 46.5% with average value of being 40.9% (SD = 0.1) from the total TB cases to its peak 66.5% in 2011 and then it decreased to 30.6% in 2008 (Table 1) (Figure 3).

Out of 4100 smear-positive TB cases, highest number of cases (1233 (23.1%)) were reported among age group ranges from 25 up to 34 followed by 374 (7%) in age group of 15 up to 24 while lowest number of cases (25(0.5%)) were reported among age group less than 4 years. Out of the total highest numbers of smear-positive pulmonary TB cases, males and females accounted 710 (13.3%) and 523 (9.8%), respectively. Numbers of smear-positive pulmonary TB cases were higher in males than females across all age groups (Table 2).

**Table 2:** Smear-positive pulmonary TB cases by sex and age groups in Gambella Regional State from 2006 up to 2011

**Age**	**Sex**		**Number (%)**
	**M (%)**	**F (%)**	
0–4	13 (0.3)	12 (0.2)	25 (0.5)
5–14	59 (1.1)	41 (0.8)	100 (1.9)
15–24	199 (3.7)	175 (3.3)	374 (7)
25–34	710 (13.3)	523 (9.8)	1233 (23.1)
35–44	162 (3)	104 (1.9)	266 (4.9)
45–54	63 (1.2)	51 (1)	114 (2.2)
55–64	40 (0.8)	32 (0.6)	72 (1.4)
65+	19 (0.4)	10 (0.2)	29 (0.6)
Total	1240 (23.2)	973 (18.2)	2213 (41.4)

NB: N = 5341(Total number of TB patients during the period).

A quarterly report format of WHO for DOTS program mainly focused on case detection and treatment outcome for smear-positive pulmonary TB, because smear-positive pulmonary TB cases were infectious and were much of a public health importance compared to the other forms of TB. Treatment outcomes were available for those patients who were treated by DOTS program from 2002 up to 2011. Treatment outcome reports of year 2012 were not included because they were a cohort for the year 2013 and need to be followed for their outcome results until next end of the year.

Similarly, a total of 3836 smear-positive pulmonary TB patients were considered as a cohort for treatment outcome. Two thousand five hundred-ninety five (67.7%) of them were evaluated for their treatment outcome from the period of 2002 up to 2011. Among the total evaluated smear-positive TB cases for treatment, 1177 (30.7%) were cured and 959 (25%) completed their treatment. Hence, treatment success rate was calculated to be 2136 (55.7%) (Table 1).

Therefore, treatment success rate (TSR) had an average value of being 55.7% (SD = 0.28) and from 2002 to 2011 with its peak value of being 93.9% in 2010 and it significantly declined in 2002 by 13.2%. The targets by WHO for treatment success rate had already been achieved as it become more than 85% from 2009 onwards in the region (Table 3) (Figure 3).

**Table 3:** Treatment outcome of smear-positive pulmonary TB cases in Gambella Regional State from 2002 up to 2011

**Year**	**Cohortcases (N)**	**Evaluated (%)**	**Cured (%)**	**Completed (%)**	**Success (%)**	**Death (%)**	**Failure (%)**	**Default (%)**	**TO (%)**
2002	273	53 (19.4)	11 (4.03)	25 (9.2)	36 (13.2)	9 (3.3)	0	2 (0.7)	6 (2.2)
2003	526	313 (59.5)	106 (20.2)	112 (21.3)	218 (41.4)	37 (7)	0	27 (5.1)	18 (3.4)
2004	313	244 (78)	109 (34.8)	65 (20.8)	174 (55.6)	23 (7.3)	3 (1)	27 (8.6)	13 (4.2)
2005	511	363 (71)	177 (34.6)	98 (19.2)	275 (53.8)	29 (5.7)	7 (1.4)	32 (6.3)	20 (3.9)
2006	640	274 (42.8)	141 (22)	75 (11.7)	216 (33.8)	15 (2.3)	0	22 (3.4)	21 (3.3)
2007	446	221 (49.6)	98 (22)	111 (24.9)	209 (46.9)	5 (1.1)	0	3 (0.7)	4 (0.9)
2008	168	168 (100)	98 (58.3)	44 (26.2)	142 (84.5)	3 (1.8)	0	1 (0.6)	22 (13.1)
2009	297	297 (100)	154 (51.9)	125 (42.1)	279 (93.9)	4 (1.3)	0	9 (3)	5 (1.7)
2010	374	374 (100)	155 (41.4)	167 (44.7)	322 (86.1)	18 (4.8)	0	31 (8.2)	0
2011	288	288 (100)	128 (44.4)	137 (47.6)	265 (92)	8 (2.8)	2 (0.7)	7 (2.4)	1 (0.3)
Total	3836	2595 (67.7)	1177 (30.7)	959 (25)	2136 (55.7)	151 (3.9)	12 (0.3)	161 (4.2)	110 (2.9)

Moreover, cure rate relatively increased from 4.03% to 44% over the study period. The same was true for TB patients who had completed their treatment which ranged from 9.2% up to 47.6%. The average value of death rate and defaulter rate were found to be 3.9%(SD = 0.02) and 4.2% (SD = 0.02), respectively. Additionally, failure rate and transfer out rate fluctuated over the years and reached to the average value of being 0.3% and 2.9%, respectively (Table 3).

## Discussion

The study showed that 10024 cases were registered with all forms of TB for treatment. Of which, 5615 (56%) were male patients and 4409 (44%) females. Similar findings were also seen in southern region of the country [10] and Addis Ababa City Administration [11]. This might be due to the fact that females were underutilized of DOTS service, and might also indicate that most of males were spent their time out side home for work than females which predisposed them for susceptibility of TB infection. This was also true for the studies done on ten year assessment of DOTS program in Oromia and Benshangul Gumuze Regional States of the country [12,13]. Additionally, another study done on barriers in accessing tuberculosis treatment in Gambian women’s showed that females used traditional healers more, probably because of stronger traditional beliefs, time constraints and increased confidentiality. In the same study, poor socio-economic condition, lack of knowledge about TB and stigma were also reported, and worst in female patients [14]. However, further studies should be needed to investigate the findings.

An increased number of males 123 (3%) who returned after default as compared females 96 (2.3%) showed in the study. A similar study conducted on unsuccessful treatment outcome for DOTS program in South India revealed that more males returned after default from treatment than females and the findings were significantly associated with patients’ age ≥ 45 years, previous history of anti-tuberculosis treatment, being male, case finding by community survey, irregularity of treatment during the intensive phase, alcoholism, type of disease and smoking [15].

The highest numbers of cases (1233 (23.1%)) were observed for smear-positive pulmonary TB among age group from 25 up to 34 followed by 374 (7%) in age group of 15 up to 24. This might be attributed to increased incidence of HIV/AIDS and population migration more than others age groups [16]. Highest numbers of smear-positive pulmonary TB cases among similar age groups were also observed by previous studies [12,13].

In the study, case detection rate of smear-positive pulmonary TB had increased from 31.7% to 46.5% from the total TB cases registered for the study period. The increment might be linked to decentralization and expansion of DOTS program. It might also be explained by the influence of referred smear-positive pulmonary TB from other region and the improvement of diagnostic settings of health facilities in the region [17]. However, the total average value of CDR was 40.9% for the study period which was less than WHO target of 70%. A similar result was obtained on the study done in Addis Ababa City Administration [11]. Hence, the possible explanation might be as the CDR relies on an estimate for the incidence of TB, it is difficult to measure accurately in most settings especially in the context of a high prevalence of HIV [18]. Additionally, the national average CDR for the year 2008 was 34% [2]. This might be due to inadequate decentralization of DOTS program, shortage of resource and trained personnel, low sensitivity of smear microscopy and high level of HIV prevalence in the region.

The study revealed that the average value of treatment success rate (55.7%) was lower than 85% of WHO target as compared to national average 84% [2] and Africa 72% [19] from 2002 up to 2008 in the region, but it showed more than 85% from 2009 onwards. Decrease in treatment success rate (less than 85%) for specified years might be due to poor observation of patients during the course of treatment, poor patient treatment compliance, poor standardized and improper recording and reporting system, inadequate treatment regimens and increase in incidence of drug resistant strains. The possible explanations for increase treatment success rate (more than 85%) from 2009 up to 2011 might be due to adequate treatment regimens, good adherence to treatment or government commitment to ensure comprehensive TB control activities [20].

The average treatment failure rate was about 0.3% for smear-positive pulmonary TB cases treated by DOTS program for the same study period. The lower failure rate could be due to the overall low prevalence of multi-drug resistant tuberculosis (MDR TB) in Ethiopia which was 1.6% for new smear positive cases [21].

## Conclusion

It is possible to achieve the recommended WHO target which is 70% of CDR for smear-positive pulmonary TB as it was significantly increased from 31.7% to 46.5% for the study period, and 85% of TSR for its treatment out come as it was already been fulfilled the targets for treatment outcomes (more than 85%) from 2009 up to 2011 in the region. However, it requires strong efforts to enhance case detection rate (40.9%) for smear-positive pulmonary TB through implementing alternative case finding strategies.

### Limitation of the study

The existence of poor recording and reporting mechanism in the study at each level of health system which comprised from health facilities up to the national program might have an effect in finding valid and reliable data for proper monitoring and evaluation of the program as the data source was secondary, which was from DOTS implementing health facilities. In addition, the expansion and the decentralization of DOTS were not available at each year in the region, except the current one and this in turn prevents the study from, for example, addressing any possible significant association either between the expansions of the program with the current case detection rate and treatment out come.

## Abbreviations

AASTU: Addis Ababa Science and Technology University; CDR: Case detection rate; CSA: Central statistical agency; DOTS: Directly observation treatment short-course; EHNRI: Ethiopian Health Nutrition and Research Institution; FMoH: Federal Ministry of Health; HBC: High burden countries; HF: Health facility; HMIS: Health management information system; HIV: Human immunodeficiency virus; MDR TB: Multi drug resistant tuberculosis; NGO: Non Governmental Organization; NTLCP: National Tuberculosis and Leprosy Control Program; PTB: Pulmonary tuberculosis; SD: Standard deviation; SPSS: Statistical package for social science; TB: Tuberculosis; TO: Transfer out; TSR: Treatment success rate; WHO: World Health Organization.

## Competing interests

The authors declare that they have no any competing interests.

## Authors’ contribution

SS, BM and DW conceived the idea: SS designed the study; SS and BM collected the data in the field and SS drafted the manuscript; All authors supervised the overall conduct of the study and SS, BM, DW interpreted the results; SS, BM, WE and DW drafted the manuscript for publication. All authors participated in the write up, read and approved the final manuscript.
